# Inclusive Composite Interval Mapping of QTL by Environment Interactions in Biparental Populations

**DOI:** 10.1371/journal.pone.0132414

**Published:** 2015-07-10

**Authors:** Shanshan Li, Jiankang Wang, Luyan Zhang

**Affiliations:** The National Key Facility for Crop Gene Resources and Genetic Improvement, Institute of Crop Science, and CIMMYT China Office, Chinese Academy of Agricultural Sciences, Beijing, China; Beijing Forestry University, CHINA

## Abstract

Identification of environment-specific QTL and stable QTL having consistent genetic effects across a wide range of environments is of great importance in plant breeding. Inclusive Composite Interval Mapping (ICIM) has been proposed for additive, dominant and epistatic QTL mapping in biparental populations for single environment. In this study, ICIM was extended to QTL by environment interaction (QEI) mapping for multi-environmental trials, where the QTL average effect and QEI effects could be properly estimated. Stepwise regression was firstly applied in each environment to identify the most significant marker variables which were then used to adjust the phenotypic values. One-dimensional scanning was then conducted on the adjusted phenotypic values across the environments in order to detect QTL with either average effect or QEI effects, or both average effect and QEI effects. In this way, the genetic background could be well controlled while the conventional interval mapping was applied. An empirical method to determine the threshold of logarithm of odds was developed, and the efficiency of the ICIM QEI mapping was demonstrated in simulated populations under different genetic models. One actual recombinant inbred line population was used to compare mapping results between QEI mapping and single-environment analysis.

## Introduction

QTL by environment interaction (QEI) widely exists in crops and other organisms. Studies on QEI contribute to the efficient use of marker-assisted selection (MAS) in breeding, and better understanding of genetic architecture of important quantitative traits and genotype by environment interactions [1,2,3]. As a consequence, many theoretical and applied studies have been conducted on QEI analysis in multi-environmental trials.

Analysis of variance (ANOVA), the simplest method, tested one marker at a time and had no background control, which gave rise to many false positive QTL [4]. Composite interval mapping was applied to detect QEI when multiple environments were regarded as multiple traits [5,6], but the effect of QTL at the current interval may be absorbed by the background variables, which resulted in biased estimation [7]. Tinker and Mather [8] proposed the simplified composite interval mapping suitable for large dataset. Approximate analysis of QEI was proposed with no limits on the number of environments [9]. Hackett et al. [10] proposed a multi-trait QTL mapping method for QTL position estimation based on the regression mapping approach of Haley and Knot [11].

Mixed model approaches have also been used in QEI detection. Wang et al. [12] developed a methodology using mixed linear models, but the results may be susceptible to the specified models [13]. Piepho [13] showed how random QEI effects and genetic correlation could be straightforwardly handled in the mixed model framework. Malosetti et al. [14] presented a strategy combining mixed model with factorial regression. A method using factorial regression and partial least squares [15] was an extension of the statistical approaches developed by Crossa et al. [16] and van Eeuwijk et al. [17]. A strategy combining mixed model, simple and composite interval mapping, and introducing environmental co-variables was developed by Boer et al. [1]. However, inconsistent mapping results were observed owing to different fixed and random effect assumptions and variance-covariance matrix choices, for example, identical genetic variation, compound symmetry, first-order analytic model, uniform covariance and heterogeneous variance and so on [1]. Further studies are needed so as to validate the efficiency of the mixed-model based methods. In addition, mixed models have high computational complexity and are much time consuming, which is less suitable for large data sets.

Recently, the method based on Bayesian model [18,19] was proposed for QEI analysis, using Markov Chain Monte Carlo. In this method, estimation of QEI was simplified by treating each marker as a putative QTL. Strategies combining composite interval mapping with additive main effects and multiplicative interaction model were proposed to decrease the noise of phenotypic values especially when the GEI noise is large [20]. Generally speaking, Bayesian model is hard to be implemented because of its computation burden and difficulty in choosing appropriate prior distributions [18,19].

Inclusive Composite Interval Mapping (ICIM) was proposed for additive, dominant and epistatic QTL mapping in biparental populations [7,21–24]. ICIM applies a two-step mapping strategy. Firstly, stepwise regression is conducted to select the significant markers for additive QTL mapping or marker-pairs for epistatic QTL mapping considering all marker information simultaneously. Secondly, the phenotypic values are adjusted by the marker variables retained in the regression equation except the two markers flanking the current scanning position(s) for background control. The adjusted phenotypic values are subsequently used in interval mapping. This strategy effectively separates the cofactor selection from the interval mapping using Maximum Likelihood (ML) method. Genetic background control decreases variance of the estimated genetic parameter, and therefore increases accuracy of estimates and the detection power [5,7,21,25]. Extensive simulations have illustrated that ICIM is an efficient mapping method with higher detection power, lower false discovery rate (FDR) and less biased estimates of QTL effect and position [7,22–24,26,27]. With the user-friendly software of QTL IciMapping (freely available from www.isbreeding.net), ICIM has been widely applied in QTL mapping researches (for examples, see [28–31]).

In this study, we focused on the additive QEI analysis. Our objectives were: (1) to propose an inclusive linear model capable of absorbing all genetic effects in QEI mapping; (2) to extend ICIM to additive QTL by environment interactions, and estimate the average effect, QEI effects and the phenotypic variance explained (PVE); (3) to propose an empirical method to determine the LOD threshold in QEI mapping; (4) to validate the proposed QEI mapping methods in simulated and an actual maize recombinant inbred lines (RIL) populations.

## Materials and Methods

### Genetic and linear regression models in QEI mapping

DH population was used to illustrate the ICIM QEI mapping. For simplicity, it is supposed that two inbred lines P_1_ and P_2_ differ at *m* QTL, being located in *m* intervals defined by *m*+1 markers on one chromosome. If no QTL is located in a marker interval, the average and interaction effects of the QTL are treated as zero. QTL genotypes of P_1_ and P_2_ are assumed to be *Q*_1_ *Q*_1_ *Q*_2_ *Q*_2_ *…Q*_*m*_ *Q*_*m*_ and *q*_1_ *q*_1_ *q*_2_ *q*_2_ *…q*_*m*_ *q*_*m*_, respectively. Suppose that a DH population is derived from the F_1_ hybrids of P_1_ and P_2_ and phenotyped in *e* environments. For each individual, **X** = (*x*_1_, *x*_2_,…, *x*_*m*_, *x*_m+1_) represents the marker variables equal to 1 or -1, standing for two marker types (i.e. P_1_ marker type and P_2_ marker type). **G** = (*g*_1_, *g*_2_,*…*, *g*_*m*_) represents QTL variables equal to 1 or -1, standing for two QTL genotypes (i.e. P_1_ QTL type and P_2_ QTL type). Let *a*_1h_, *a*_2h_,*…*, *a*_*mh*_ represent the additive effects of the *m* QTL in the *h* ^th^ environment, respectively. Under the assumption of additivity of QTL effects, *G*_*h*_, the genotypic value of an individual in the *h* ^th^ environment under the additive genetic model, can be written in Eq (1).

$$G_{h}=\mu_{h}+\overset{m}{\underset{j=1}{\sum }}a_{jh}g_{j}\;(h=1,\cdots ,e).$$ (1)

The expected frequency of the *j* ^th^ QTL genotype depends on its position at the chromosomal interval flanked by the *j* ^th^ and (*j*+1)^th^ markers and the length of this interval [7,23,32,33,], i.e.,

$$E(g_{j}|X)=\lambda_{j}x_{j}+\rho_{j}x_{j+1},$$ (2)

where *λ*_*j*_ and *ρ*_*j*_ are functions of the three recombination fractions between the *j* ^th^ marker and *j* ^th^ QTL, between the *j* ^th^ QTL and (*j*+1)^th^ marker, and between the *j* ^th^ and (*j*+1)^th^ markers. The expectation of genotypic value *G*_*h*_ conditional on marker type **X** can be denoted as,

$$E(G_{h}|X)=\mu_{h}+\overset{m}{\underset{j=1}{\sum }}a_{jh}(\lambda_{j}x_{j}+\rho_{j}x_{j+1})\hat{=}b_{0h}+\overset{m+1}{\underset{j=1}{\sum }}b_{jh}x_{j},$$ (3)

where *b*_0h_ = *μ*_*h*_, *b*_1h_ = *λ*_1_ *a*_1h_, and *b*_*jh*_ = *ρ*_j-1_ *a*_(j-1)h_ *+λ*_*j*_ *a*_*jh*_(*j* = 2,…,m). The coefficient of the *j* ^th^ marker in the *h* ^th^ environment, i.e. *b*_*jh*_, is only affected by QTL in the (*j*-1)^th^ and *j* ^th^ marker intervals. Therefore, when QTL are isolated by at least one blank interval, *b*_*jh*_ and *b*_(j+1)h_ contain all the position and additive effect information of QTL in the *j* ^th^ interval. These statistical properties provide the theoretical basis of QEI mapping.

Suppose a DH mapping population has observations on a quantitative trait of interest and genotyping information on *m*+1 ordered markers. The following linear regression model can be used in the additive QEI mapping, i.e.,

$$y_{ih}=b_{0h}+\overset{m+1}{\underset{j=1}{\sum }}b_{jh}x_{ij}+\varepsilon_{ih},$$ (4)

where *y*_*ih*_ is the phenotypic value of the *i* ^th^ individual in the *h* ^th^ environment; *b*_0h_ is the overall mean of linear model in the *h* ^th^ environment; *x*_*ij*_ is the indicating variable for the *j* ^th^ marker’s genotype of the *i* ^th^ individual, which is equal to 1 or -1 standing for P_1_ type or P_2_ type respectively; *b*_*jh*_ is the partial regression coefficient of phenotype on the *j* ^th^ marker in the *h* ^th^ environment; and *ε*_*ih*_ is the residual random error in the *h* ^th^ environment that is assumed to be normally distributed. Stepwise regression can be therefore conducted for phenotypic value in each environment to select significant markers, similar to additive mapping in single environment [7].

### One-dimensional scanning of QEI mapping

For a testing position in the *k* ^th^ marker interval, the phenotypic value of the *i* ^th^ individual in the *h* ^th^ environment was adjusted by

$$\Delta y_{ih}=y_{ih}-\underset{j\neq k,k+1}{\sum }{\hat{b}}_{jh}x_{ij},$$ (5)

where ${\hat{b}}_{jh}$ is the estimate of *b*_*jh*_ of significant markers selected by stepwise regression in model (4). The phenotypic value Δ*y*_*ih*_ contains QTL information in the current interval and does not change until the testing position moves to the next interval. Traditional interval mapping was conducted on the adjusted phenotypic values given by Eq (5).

For a testing position in an interval, individuals of DH population can be classified into four groups based on the types of the two flanking markers. If there is one QTL (with the two alleles denoted as *Q* and *q*) at the current testing position, each marker group has both QTL genotypes *QQ* and *qq*, and hence follows a mixture distribution of two components $N(\mu_{1h},\sigma_{\varepsilon h}^{2})$ and $N(\mu_{2h},\sigma_{\varepsilon h}^{2})$. In each marker group, frequencies of the two QTL genotypes depend on the recombination fractions between the putative QTL and two flanking markers, and they are different for the four marker groups. Existence of QTL at the current scanning position can be tested by the following hypotheses:

$$H_{0}:\;\mu_{1h}=\mu_{2h},\text{ i.e., }\bar{a}=0\text{ and }ae_{h}=0\text{ (}h=1,\cdots ,e\text{);}$$

$$H_{1}:\text{ non-}H_{0}\text{, i.e., for at least one environment, }\mu_{1h}\neq \mu_{2h};$$

$$H_{2}:\text{ non-}H_{0}\text{, but }\bar{a}=0;$$

where $\bar{a}$ is the average effect of the putative QTL across the environments, i.e., $\;\bar{a}=\frac{1}{e}\sum_{h=1}^{e}a_{h}$, and $\;ae_{h}=a_{h}-\bar{a}$ is the QEI effect in the *h* ^th^ environment. In *H*_1_, $\;\bar{a}\neq 0$, or *ae*_*h*_ *≠* 0 for at least one environment. Therefore, the likelihood ratio of hypotheses *H*_1_ versus *H*_0_ can be used to test all effects, i.e. both the average effect and QEI effects. In *H*_2_, $\;\bar{a}=0$, but *ae*_*h*_ *≠* 0 for at least one environment. Therefore, *H*_2_ is nested into *H*_1_ by adding the condition $\;\bar{a}=0$. Size of the average effect makes the difference between *H*_1_ and *H*_2_. The likelihood ratio of hypotheses *H*_1_ versus *H*_2_ can test the significance of average effects. Difference between likelihood functions of *H*_2_ and *H*_0_ came from the QEI effects, as both of them have the restriction $\bar{a}=0$. Therefore, the likelihood ratio of hypotheses *H*_2_ versus *H*_0_ can test the significance of QEI effects.

The log-likelihood function under the alternative hypothesis *H*_1_ is,

$$L_{1}=\overset{e}{\underset{h=1}{\sum }}\overset{4}{\underset{l=1}{\sum }}\underset{i\in S_{l}}{\sum }\text{log}[\pi_{l1}f(\Delta y_{ih};\mu_{1h},\sigma_{\varepsilon h}^{2})+\pi_{l2}f(\Delta y_{ih};\mu_{2h},\sigma_{\varepsilon h}^{2})],$$ (6)

where *S*_*l*_ (*l* = 1, 2, 3, and 4) denotes the *l* ^th^ marker type group; *π*_*l1*_ and *π*_*l2*_ are the proportions of two QTL genotypes *QQ* and *qq* in the *l* ^th^ group, respectively; $f(\cdot \;;\mu_{kh},\sigma_{\varepsilon h}^{2})$ represents the density of the *k* ^th^ normal distribution in the *h* ^th^ environment.

The expectation and conditional maximization (ECM) algorithm [34] was used to estimate the two means and one variance in the *h* ^th^ environment in Eq (6). Their initial values can be determined by:

$$\mu_{1h}^{\left(0\right)}=\frac{1}{n_{1}}\sum_{i=1}^{n_{1}}\Delta y_{ih},\;\mu_{2h}^{\left(0\right)}=\frac{1}{n_{4}}\sum_{i=n_{1:3}+1}^{n}\Delta y_{ih},\text{ and}$$

$$\sigma_{\varepsilon h}^{2(0)}=\frac{1}{n_{1}+n_{4}}\left[\overset{n_{1}}{\underset{i=1}{\sum }}{\left(\Delta y_{ih}-\mu_{1h}^{\left(0\right)}\right)}^{2}+\overset{n}{\underset{i=n_{1:3}+1}{\sum }}{\left(\Delta y_{ih}-\mu_{2h}^{\left(0\right)}\right)}^{2}\right]\;(h=1,\cdots ,e),$$

where *n*_1:3_ is the summation of *n*_1_ to *n*_3_. In the E-step, the posterior probability of the *i* ^th^ individual (*i* = 1, …, *n*_*h*_; *h* = 1, …, *e*) belonging to the *k* ^th^ QTL genotype (*k* = 1, 2) was calculated as,

$$w_{ik}^{\left(0\right)}=\pi_{lk}\overset{e}{\underset{h=1}{\sum }}f(\Delta y_{ih};\mu_{kh}^{\left(0\right)},\sigma_{\varepsilon h}^{2(0)})/\left[\pi_{l1}\overset{e}{\underset{h=1}{\sum }}f(\Delta y_{ih};\mu_{1h}^{\left(0\right)},\sigma_{\varepsilon h}^{2(0)})+\pi_{l2}\overset{e}{\underset{h=1}{\sum }}f(\Delta y_{ih};\mu_{2h}^{\left(0\right)},\sigma_{\varepsilon h}^{2(0)})\right],$$

where *l* denoted the marker group into which the *i* ^th^ individual was classified. In the M-step, the three groups of parameters were updated as,

$$\mu_{kh}^{\left(1\right)}=\overset{n}{\underset{i=1}{\sum }}w_{ik}^{\left(0\right)}\Delta y_{ih}/\overset{n}{\underset{i=1}{\sum }}w_{ik}^{\left(0\right)}\;(k=1,2),\text{ and }\sigma_{\varepsilon h}^{2(1)}=\frac{1}{n}\overset{n}{\underset{i=1}{\sum }}\overset{2}{\underset{k=1}{\sum }}w_{ik}^{\left(0\right)}{\left(\Delta y_{ih}-\mu_{kh}^{\left(1\right)}\right)}^{2}.$$

The EM algorithm continued until the difference in the likelihood between two consecutive iterations reached a pre-assigned precision, say 10^−6^. The ML estimates thus obtained were represented by ${\hat{\mu }}_{1h}$, ${\hat{\mu }}_{2h}$ and ${\hat{\sigma }}_{\varepsilon h}^{2}$. Then the additive effect under the *h* ^th^ environment was calculated as $a_{h}=\frac{1}{2}({\hat{\mu }}_{1h}-{\hat{\mu }}_{2h})$.

The log-likelihood function under the alternative hypothesis *H*_2_ is,

$$L_{2}=\overset{e}{\underset{h=1}{\sum }}\overset{4}{\underset{l=1}{\sum }}\underset{i\in S_{l}}{\sum }\text{log}[\pi_{l1}f(\Delta y_{ih};\mu_{1h},\sigma_{\varepsilon h}^{2})+\pi_{l2}f(\Delta y_{ih};\mu_{2h},\sigma_{\varepsilon h}^{2})]-\lambda \bar{a},$$

where *λ* is the Lagrange multiplier.

In the EM algorithm for *L*_2_, the calculation of posterior probability was the same as previous one. In the M-step, the three parameters were updated as follows,

$$\mu_{1h}^{\left(1\right)}=\left[\overset{n}{\underset{i=1}{\sum }}w_{i1}^{\left(0\right)}\Delta y_{ih}-\frac{1}{2e}\sigma_{\varepsilon h}^{2}\lambda \right]/\overset{n}{\underset{i=1}{\sum }}w_{i1}^{\left(0\right)},$$

$$\mu_{2h}^{\left(1\right)}=\left[\sum_{i=1}^{n}w_{i2}^{\left(0\right)}\Delta y_{ih}+\frac{1}{2e}\sigma_{\varepsilon h}^{2}\lambda \right]/\sum_{i=1}^{n}w_{i2}^{\left(0\right)}\text{ and}$$

$$\sigma_{\varepsilon h}^{2(1)}=\frac{1}{n}\overset{n}{\underset{i=1}{\sum }}\overset{2}{\underset{k=1}{\sum }}w_{ik}^{\left(0\right)}{\left(\Delta y_{ih}-\mu_{kh}^{\left(1\right)}\right)}^{2},$$

where $\lambda =\frac{2e}{\left(\frac{1}{\overset{n}{\underset{i=1}{\sum^{\text{​}}}}w_{i1}}+\frac{1}{\overset{n}{\underset{i=1}{\sum^{\text{​}}}}w_{i2}}\right)\overset{e}{\underset{h=1}{\sum^{\text{​}}}}\sigma_{\varepsilon h}^{2}}\overset{e}{\underset{h=1}{\sum^{\text{​}}}}\left(\frac{\overset{n}{\underset{i=1}{\sum^{\text{​}}}}\Delta y_{ih}w_{i1}}{\overset{n}{\underset{i=1}{\sum^{\text{​}}}}w_{i1}}-\frac{\overset{n}{\underset{i=1}{\sum^{\text{​}}}}\Delta y_{ih}w_{i2}}{\overset{n}{\underset{i=1}{\sum^{\text{​}}}}w_{i\text{2}}}\right).$

Under the null hypothesis *H*_0_, the Δ*y*_*ih*_ follow the normal distribution of $N(\mu_{0h},\sigma_{0h}^{2})$. The mean and variance of this distribution can be estimated as,

$${\hat{\mu }}_{0h}=\frac{1}{n}\overset{n}{\underset{i=1}{\sum }}\Delta y_{ih}\text{, and }{\hat{\sigma }}_{0h}^{2}=\frac{1}{n}\overset{n}{\underset{i=1}{\sum }}{\left(\Delta y_{ih}-{\hat{\mu }}_{0h}\right)}^{2}.$$

The log-likelihood function under the null hypothesis *H*_0_ is,

$$L_{0}=\overset{e}{\underset{h=1}{\sum }}\overset{n}{\underset{i=1}{\sum }}\text{log}[f(\Delta y_{ih};\mu_{0h},\sigma_{0h}^{2})].$$

The LOD score (denoted by LOD_A_) calculated by *L*_1_-*L*_2_ indicates whether there is significant average effect at the testing position. The LOD score (denoted by LOD_AE_) calculated by *L*_2_-*L*_0_ indicates whether there are significant QEI effects. Sum of LOD_A_ and LOD_AE_ (denoted by LOD) gives the overall test statistic indicating the significance of both average effect and QEI effects.

### Phenotypic variation explained (PVE) by the identified QTL

In the QTL IciMapping software, PVE of additive and QEI effects at each scanning position were estimated by posterior probability *w*_*ik*_ and two QTL genotypic means ${\hat{\mu }}_{kh}\text{ (}k=\text{1, and 2)}$, which have been estimated by EM algorithm previously. For illustration, we assume there is one QTL at the current scanning position, and the expected frequency of the *k* ^th^ QTL genotype (*QQ* or *qq*) in the *h* ^th^ environment is *f*_*kh*_, (*k* = 1 and 2, and *h* = 1,…, *e*). The marginal frequency of QTL genotype is defined as the sum of QTL genotype frequencies in all environments, i.e., $f_{k\cdot }=\sum_{h=1}^{e}f_{kh}$, which can be estimated by ${\hat{f}}_{k\cdot }=\frac{1}{n}\sum_{i=1}^{n}w_{ik}$. QTL genotype is independent of environment, therefore *f*_*kh*_ can be estimated by ${\hat{f}}_{kh}=\frac{1}{e}{\hat{f}}_{k\cdot }$,. Genetic variance and QEI variance can be calculated from Table 1, a two-way table of two QTL genotypic means and a set of environments.

**Table 1:** Means and effects of QTL genotypes in multi-environmental trials.

QTL genotype	Environment					Genotypic mean	Genotypic effect
	E_1_	…	E_*h*_	…	E_*e*_		
*QQ*	${\hat{\mu }}_{11}$	…	${\hat{\mu }}_{1h}$	…	${\hat{\mu }}_{1e}$	${\hat{\mu }}_{1\cdot }$	${\hat{\mu }}_{1\cdot }-{\hat{\mu }}_{\cdot \cdot }$
*qq*	${\hat{\mu }}_{21}$	…	${\hat{\mu }}_{2h}$	…	${\hat{\mu }}_{2e}$	${\hat{\mu }}_{2\cdot }$	${\hat{\mu }}_{2\cdot }-{\hat{\mu }}_{\cdot \cdot }$
Environmental mean	${\hat{\mu }}_{\cdot 1}$	…	${\hat{\mu }}_{\cdot h}$	…	${\hat{\mu }}_{\cdot e}$	${\hat{\mu }}_{\cdot \cdot }$(grand or overall mean)	
Environmental effect	${\hat{\mu }}_{\cdot 1}-{\hat{\mu }}_{\cdot \cdot }$	…	${\hat{\mu }}_{\cdot h}-{\hat{\mu }}_{\cdot \cdot }$	…	${\hat{\mu }}_{\cdot e}-{\hat{\mu }}_{\cdot \cdot }$		

The grand mean and two weighted means were calculated as, ${\hat{\mu }}_{\cdot \cdot }=\sum_{k=1}^{2}\sum_{h=1}^{e}f_{kh}{\hat{\mu }}_{kh}$, and ${\hat{\mu }}_{k\cdot }=\sum_{h=1}^{e}f_{\cdot h}{\hat{\mu }}_{kh}=\frac{1}{e}\sum_{h=1}^{e}{\hat{\mu }}_{kh}$ and ${\hat{\mu }}_{\cdot h}=\sum_{k=1}^{2}f_{k\cdot }{\hat{\mu }}_{kh}$, respectively, where *f*_*kh*_ is the frequency of the *k* ^th^ QTL genotype (*QQ* or *qq*) in the *h* ^th^ environment; $f_{\cdot h}=\frac{1}{e}$ is the frequency of *h* ^th^ environment; *f*_*k*._ is the frequency of the *k* ^th^ QTL genotype.

The deviation between QTL genotypic mean and the grand mean can be decomposed into three components, i.e.,

$${\hat{\mu }}_{kh}-{\hat{\mu }}_{\cdot \cdot }=({\hat{\mu }}_{k\cdot }-{\hat{\mu }}_{\cdot \cdot })+({\hat{\mu }}_{\cdot h}-{\hat{\mu }}_{\cdot \cdot })+({\hat{\mu }}_{kh}-{\hat{\mu }}_{k\cdot }-{\hat{\mu }}_{\cdot h}+{\hat{\mu }}_{\cdot \cdot }).$$

The three components stood for QTL average effect, environmental effect and QEI effect, respectively. In statistics, it can be proved the decomposition is orthogonal. In DH population, genetic variance is equal to the additive variance, which can be calculated as,

$$V_{A}=\overset{2}{\underset{k=1}{\sum }}f_{k\cdot }{\left({\hat{\mu }}_{k\cdot }-{\hat{\mu }}_{\cdot \cdot }\right)}^{2}=4f_{1\cdot }f_{2\cdot }{\bar{a}}^{2}.$$

QEI effect and variance can be calculated as,

$$QEI_{kh}={\hat{\mu }}_{kh}-{\hat{\mu }}_{k\cdot }-{\hat{\mu }}_{\cdot h}+{\hat{\mu }}_{\cdot \cdot }\text{ and }V_{AE}=\overset{2}{\underset{k=1}{\sum }}\overset{e}{\underset{h=1}{\sum }}f_{kh}QEI_{kh}^{2}=\frac{4}{e}f_{1\cdot }f_{2\cdot }\overset{e}{\underset{h=1}{\sum }}{\left(a_{h}-\bar{a}\right)}^{2}.$$

Then, *PVE*_*A*_ and *PVE*_*AE*_ can be calculated from $PVE_{A}=\frac{V_{A}}{V_{P}}$ and $PVE_{AE}=\frac{V_{AE}}{V_{P}}$, respectively, where *V*_*P*_ was the average of phenotypic variances in the *e* environments. Take DH population and four environments as an example. Assume the expected marginal frequencies of QTL genotypes *QQ* and *qq* are 0.4 and 0.6, which indicated the frequencies of the two genotypes in each environment were 0.1 and 0.15, respectively. Phenotypic variances in the four environments were set at 30, 20, 10 and 40 respectively, and the values of ${\hat{\mu }}_{kh}$ were given in Table 2. *PVE*_*A*_ and *PVE*_*AE*_ can be calculated as follows,

$$V_{A}=\overset{2}{\underset{k=1}{\sum }}f_{k\cdot }{\left({\hat{\mu }}_{k\cdot }-{\hat{\mu }}_{\cdot \cdot }\right)}^{2}=0.4\times 0.3^{2}+0.6\times {\left(-0.2\right)}^{2}=0.06,$$

$$V_{AE}=\overset{2}{\underset{k=1}{\sum }}\overset{4}{\underset{h=1}{\sum }}f_{kh}QEI_{kh}^{2}=3.18\text{, and }V_{P}=\frac{1}{e}\overset{e}{\underset{h=1}{\sum }}V_{Ph}=\frac{1}{4}\times (30+20+10+40\text{)}=25;$$

$$PVE_{A}=\frac{0.06}{25}=0.24\%\text{, and }PVE_{AE}=\frac{3.18}{25}=12.72\%.$$

**Table 2:** Means and effects of QTL genotypes in a DH population.

QTL genotype	Environment				Genotypic mean	Genotypic effect
	E_1_	E_2_	E_3_	E_4_		
*QQ*	14	10	12	16	13	0.3
*qq*	12	10	17	11	12.5	-0.2
Environmental mean	12.8	10	15	13	12.7	
Environmental effect	0.1	-2.7	2.3	0.3		

### Empirical formula of LOD threshold in QEI mapping

In single-environment analysis, when the null hypothesis is true, likelihood ratio test (LRT) at each scanning position follows the *χ* ^2^ distribution with the degree of freedom (*df*) equal to the number of genetic parameters be estimated in the genetic population [32,35]. Sun et al. [36] found that the number of independent tests (denoted as *M*_*eff*_) was proportional to the genome length in one-dimensional QTL scanning, and the proportion varied with marker density and population type. So *M*_*eff*_ can be estimated as the product of proportion efficient and the genome length. Let *α*_*g*_ be the genome-wide type-I error, the type-I error at each testing position should be $\alpha_{p}=\frac{\alpha_{g}}{M_{eff}}$ based on the Bonferroni correction. Therefore, the empirical LOD threshold can be determined by formula $LOD=\chi_{\alpha_{p}}^{2}(\lambda )/2ln(10)$, where $\chi_{\alpha_{p}}^{2}(\lambda )$ is the inverse *χ* ^2^ distribution that returns the critical value of a right-tailed probability *α*_*p*_ for the degree of freedom *λ*. In QTL mapping, *λ* is equal to the number of genetic parameters to be estimated [36].

This formula can also be used to determine the LOD threshold in QEI mapping by considering the difference in degree of freedom. In QEI mapping, each QTL genotype has its own distribution in each environment, and the number of independent genetic parameters to be estimated is the sum of parameters in each environment. In other words, *λ* is equal to *e* for BC_1_ (or DH and RIL) population and 2*e* for F_2_ population. For validation, LOD threshold was determined in simulated BC_1_ and F_2_ populations under null genetic model (S1 and S2 Files). The genomic information and mapping parameters were the same as unlinked and linked QTL models (to be described in the next section), except that there was no QTL located on the genome. LOD thresholds for *α*_*g*_ = 0.05 and *α*_*g*_ = 0.01 were estimated at the 95^th^ and 99^th^ percentiles of the 1000 maximum LOD scores out of 1000 runs.

### Putative genetic models in simulation studies

QTL IciMapping is integrated software for linkage map construction and QTL detection. QEI mapping has been implemented in version 4.0 of the software as the MET functionality [37]. In this study, unlinked and linked QTL models were both considered to evaluate the efficiency of QEI mapping. The genome consisted of six chromosomes, each of 150 cM in length with 16 evenly distributed markers. Two environments were considered with equal heritability in the broad sense in both models. In the unlinked QTL model, five QTL were located on five chromosomes, and the broad sense heritability was 0.5 for both environments. QTL additive effects in the two environments were given in Table 3, representing three QEI levels, i.e., strong interaction (Q2), environment-specific interaction (Q3 and Q4) and no interaction (Q1 and Q5).

**Table 3:** Predefined chromosomal positions and additive effects of five unlinked QTL.

QTL	Chr.	Pos. (cM)	A	AE_1_	AE_2_	PVE_A_ (%)	PVE_AE_ (%)	PVE (%)
Q1	1	16	0.5	0	0	12.5	0	12.5
Q2	2	3	0	0.5	-0.5	0	12.5	12.5
Q3	3	33	0.25	-0.25	0.25	3.13	3.13	6.25
Q4	4	26	0.25	0.25	-0.25	3.13	3.13	6.25
Q5	5	35	0.5	0	0	12.5	0	12.5

A, AE_1_ and AE_2_ represent average additive effect, QEI effect in E_1_ and QEI effect in E_2_ respectively. PVE, the percentage of variance explained by individual QTL, was calculated under the assumption that the frequencies of two QTL genotypes *QQ* and *qq* are equal to 0.5.

Eight QTL effect scenarios were considered for two linked QTL (Table 4), i.e., Q1 and Q2, located at 25 and 55 cM on chromosome 1. These scenarios represented different QEI levels and linkage phases (coupling or repulsion phases). For example, Q1 and Q2 had strong QEI, and they were linked in the coupling phase in model L3, and in the repulsion phase in model L7. Three levels of heritability were considered, i.e., *H* ^2^ = 0.1, *H* ^2^ = 0.5 and *H* ^2^ = 0.8.

**Table 4:** Predefined chromosomal positions and additive effects of two linked QTL.

Effect model	L1		L2		L3		L4		L5		L6		L7		L8	
QTL	Q1	Q2	Q1	Q2	Q1	Q2	Q1	Q2	Q1	Q2	Q1	Q2	Q1	Q2	Q1	Q2
Effect in E_1_	0.5	0.5	0.5	0.5	0.5	0.5	0.5	0.5	0.5	0.5	0.5	0.5	0.5	-0.5	0.5	0
Effect in E_2_	-0.5	0	-0.5	0.5	-0.5	-0.5	0	0.5	0	0	0.5	0.5	-0.5	0.5	-0.5	0.5
PVE (%) (*H* ^2^ = 0.1)	4.88	2.44	5.00	5.00	3.23	3.23	2.44	4.88	2.86	2.86	3.23	3.23	11.08	11.08	10.51	2.63
PVE (%) (*H* ^2^ = 0.5)	24.40	12.20	25.00	25.00	16.14	16.14	12.20	24.40	9.81	9.81	16.14	16.14	55.41	55.41	52.57	13.14
PVE (%) (*H* ^2^ = 0.8)	39.05	19.52	40.00	40.00	25.83	25.83	19.52	39.05	12.70	12.70	25.83	25.83	88.65	88.65	84.10	21.03

PVE, the percentage of variance explained by individual QTL, was calculated under the assumption that the frequency of genotype *QQ* is equal to 0.5.

One thousand DH populations, each of a size of 200, were generated for unlinked model (S3 File) and for each effect scenario of the two linked QTL under each heritability level (S4–S6 Files). The LOD threshold was set at 3.11 by empirical formula to ensure the genome-wide Type-I error rate (*α*_*g*_) to be less than 0.05. The scanning step was set at 1 cM. The two probabilities for entering and removing variables in stepwise regression were set at 0.001 and 0.002.

Detection power and FDR were used to evaluate the efficiency of QEI mapping. Each predefined QTL was assigned to a support interval of 10 cM centered at the predefined location. Power of each QTL was calculated as the percentage of the simulation runs having significant peaks higher than the LOD threshold in its support interval. QTL identified but out of this interval were treated as false positives. FDR was calculated as the ratio of the number of false positives to the total number of significant discovery [26,38]. For each genetic model, estimated positions and effects were calculated as the average values of all detected QTL.

### One RIL population in maize

The actual maize population used in this study was derived from a cross between inbred parents CML444 and SC-Malawi, consisting of 236 RILs [39]. A subset of 160 markers with an average missing rate of 5.12% was used to build the linkage map. Total genome length was 2105.6 cM by Haldane mapping function, and the average marker density was 13.16 cM. Days of male flowering (MFLW) were investigated in seven environments, i.e., water-stress conditions in Mexico (WSM, in 2003 and 2004) and Zimbabwe (WSZ, in 2003 and 2004), and well-watered conditions in Mexico (WWM, in 2003 and 2004) and Zimbabwe (WWZ, in 2004), which were named as WSM1, WSM2, WSZ1, WSZ2, WWM1, WWM2, and WWZ2, respectively. The average MFLW (d) in environments WSM, WSZ, WWM and WWZ were 104.2, 121.1, 65.4 and 75.6 for parental line CML444, 101.1, 114.1, 64.5 and 74.9 for parental line SC-Malawi, and 101.1, 117.3, 64.1 and 75.5 for the RIL population. Single-environment analysis and QEI mapping were conducted by QTL IciMapping 4.0 [37] (S7 File). LOD thresholds were set at 3.0 and 5.67 for single-environment analysis and QEI mapping, respectively, same as Messmer et al. [39]. To compare with the empirical LOD threshold values, permutation tests were conducted on the maize population for 1000 times [40].

## Results

### Empirical LOD threshold in QEI mapping

**Fig 1:**
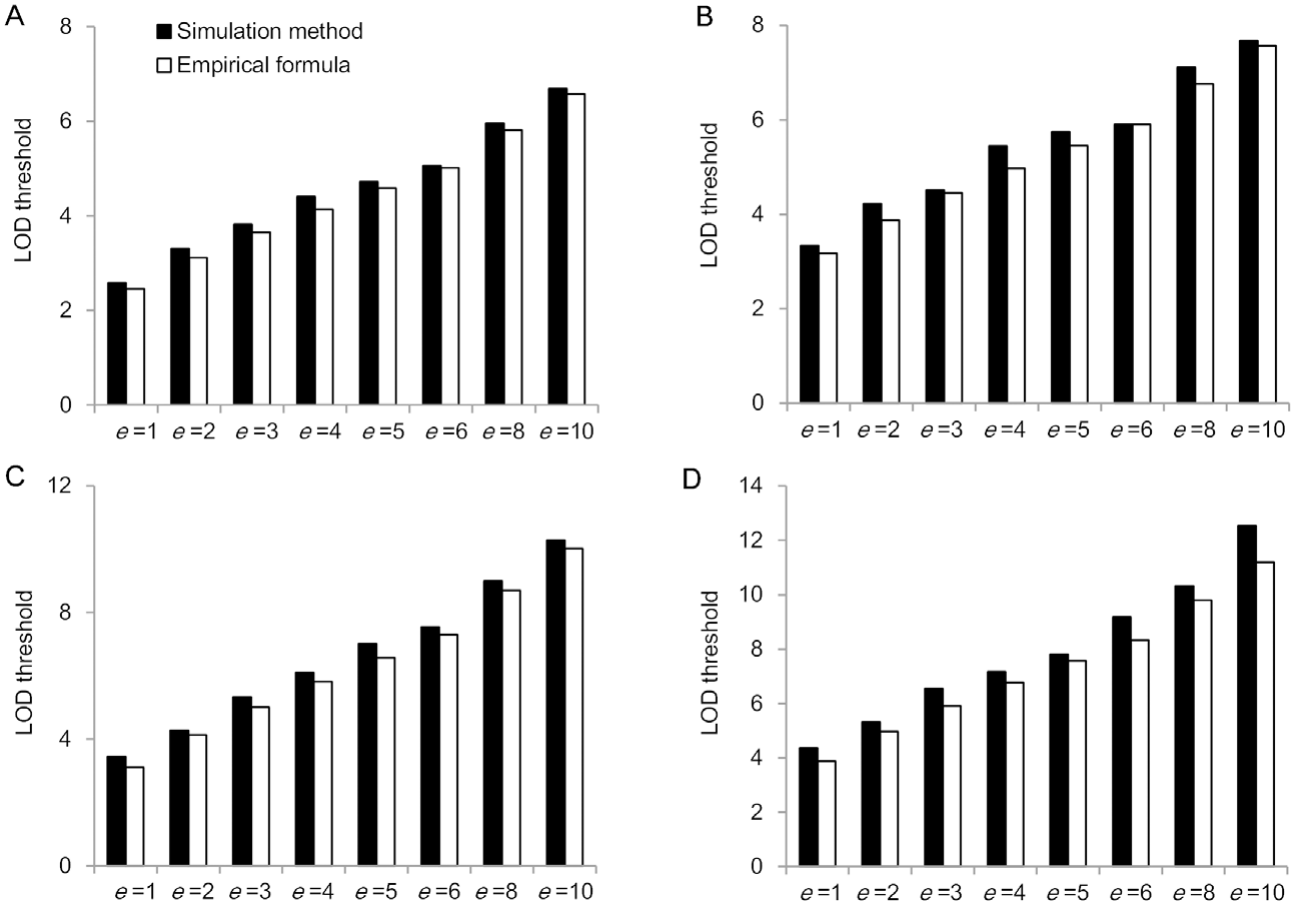
LOD thresholds of QEI mapping based on empirical formula and simulation method. (A) BC_1_ population and *α*_*g*_ = 0.05. (B) BC_1_ population and *α*_*g*_ = 0.01. (C) F_2_ population and *α*_*g*_ = 0.05. (D) F_2_ population and *α*_*g*_ = 0.01.

Fig 1 showed the LOD thresholds based on empirical formula and simulation method. Obviously, higher LOD threshold can be seen by the increase in the environment number, and the two methods resulted in similar LOD thresholds under the same significance level for both populations (i.e. BC_1_ and F_2_ populations). Thus, the empirical formula from individual environments [36] is also suitable for QEI mapping in multi-environments, considering the change in degree of freedom.

As for the RIL population, accumulated recombination frequency (represented by *R*) is much larger than the one-meiosis recombination frequency (represented by *r*) due to the continuous self-pollinations. Their relationship is well known as $R=\frac{2r}{1+2r}$, indicating *R* is approximately two times of *r*, when *r* is small. The larger recombination frequency estimated in RIL population is equivalent to a longer genome. When map distance is 1 cM in DH population, *r* and *R* are equal to 0.0099 and 0.0194 under the Haldane mapping function. The corresponding map distance is 1.98 cM for the accumulated recombination frequency in RIL population. Therefore 1 cM in DH population is expanded by 1.98 times in RIL population. Similarly, 5 cM and 10 cM are expanded by 1.91 and 1.83 times. In most genetic populations, marker density is around 5 to 10 cM, and the genome size is expanded by about 1.9 times. Therefore, the genome length should be multiplied by 1.9 before applying the LOD threshold empirical formula to RIL population.

When marker density is 10 cM, the number of independent tests is about 0.072 (*α*_*g*_ = 0.05) or 0.084 (*α*_*g*_ = 0.01) times the genome size [36]. Empirical LOD thresholds for common genome lengths were given in Table 5, by which empirical LOD thresholds can be found and applied. For example, one population is planted in four environments, the genome length is 1000 cM, and the average marker density is 10 cM. So *df* is equal to 4 for BC_1_, DH or RIL populations, and 8 for F_2_ population. According to Table 5, LOD threshold should be 4.19 for BC_1_ and DH populations, 5.87 for F_2_ population, and around 4.50 for RIL population (referring to length of 1900 cM which is between 1800 cM and 2000 cM) and *α*_*g*_ = 0.05. For the actual maize population, “a QTL was considered to be significant (comparison-wise Type-I error rate *α*_*c*_ = 0.001, experiment-wise error rate *α*_*e*_ = 0.02) when the LOD exceeded the appropriate threshold 5.67 (joint QTL, seven experiments)” in Messmer et al. [39]. LOD thresholds 6.08 and 6.89 were achieved under *α*_*g*_ = 0.05 and *α*_*g*_ = 0.01 by 1000 permutation tests. According to the empirical formula, the LOD threshold were 6.20 and 6.05 for marker densities of 10 and 20 cM under *α*_*g*_ = 0.05, and 7.11 and 7.09 under *α*_*g*_ = 0.01 for marker density of 10 and 20 cM respectively. The marker density of this population was about 13.6 cM, and the empirical LOD threshold should be around 6.1 under *α*_*g*_ = 0.05 and 7.1 under *α*_*g*_ = 0.01, close to that by permutation tests and Messmer et al. [39].

**Table 5:** Empirical LOD thresholds in QEI mapping.

Genome length (cM)	*α*_*g*_ = 0.05							*α*_*g*_ = 0.01						
	*M*_*eff*_	*df* = 1	*df* = 2	*df* = 4	*df* = 6	*df* = 8	*df* = 10	*M*_*eff*_	*df* = 1	*df* = 2	*df* = 4	*df* = 6	*df* = 8	*df* = 10
200	14	1.85	2.46	3.41	4.22	4.98	5.69	17	2.56	3.23	4.26	5.14	5.96	6.72
400	29	2.13	2.76	3.74	4.59	5.37	6.10	34	2.84	3.53	4.59	5.50	6.33	7.12
600	43	2.29	2.94	3.94	4.80	5.59	6.34	50	3.01	3.70	4.78	5.70	6.55	7.34
800	58	2.41	3.06	4.08	4.95	5.75	6.51	67	3.12	3.83	4.92	5.85	6.70	7.50
1000	72	2.50	3.16	4.19	5.07	5.87	6.64	84	2.56	3.23	4.26	5.14	5.96	6.72
1200	86	2.57	3.24	4.27	5.16	5.97	6.74	101	3.29	4.00	5.11	6.05	6.92	7.73
1400	101	2.63	3.30	4.35	5.24	6.06	6.83	118	3.35	4.07	5.18	6.13	7.00	7.81
1600	115	2.69	3.36	4.41	5.31	6.13	6.90	134	3.41	4.13	5.24	6.20	7.07	7.88
1800	130	2.74	3.41	4.47	5.37	6.19	6.97	151	3.46	4.18	5.30	6.26	7.13	7.95
2000	144	2.78	3.46	4.52	5.42	6.25	7.03	168	3.50	4.23	5.35	6.31	7.18	8.01
2200	158	2.82	3.50	4.56	5.47	6.30	7.08	185	3.54	4.27	5.39	6.36	7.23	8.06
2400	173	2.85	3.54	4.60	5.51	6.35	7.13	202	3.57	4.30	5.44	6.40	7.28	8.11
2600	187	2.89	3.57	4.64	5.55	6.39	7.18	218	3.61	4.34	5.47	6.44	7.32	8.15
2800	202	2.92	3.61	4.68	5.59	6.43	7.22	235	3.64	4.37	5.51	6.48	7.36	8.19
3000	216	2.94	3.64	4.71	5.63	6.47	7.26	252	3.67	4.40	5.54	6.51	7.40	8.23
3500	252	3.01	3.70	4.78	5.70	6.55	7.34	294	3.73	4.47	5.61	6.59	7.48	8.31
4000	288	3.06	3.76	4.85	5.77	6.62	7.42	336	3.79	4.53	5.67	6.65	7.54	8.38

LOD thresholds shown in the table were calculated under a marker density of 10 cM. The proportion coefficients of *M*_*eff*_ and genome length are 0.072 and 0.084 when *α*_*g*_ is 0.05 and 0.01.

### Power analysis for the unlinked QTL model

**Fig 2:**
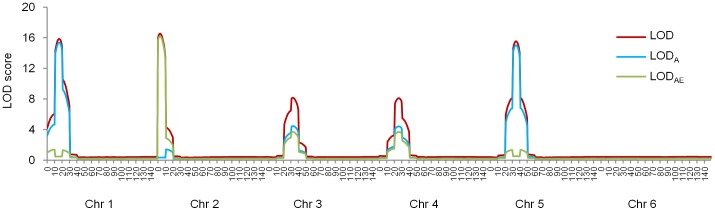
Average LOD profiles across 1000 simulation runs for the unlinked QTL model. LOD, LOD_A_ and LOD_AE_ are LOD scores for detecting QTL with both average and QEI effects, QTL only with average effect, and QTL only with QEI effects, respectively.

Fig 2 displayed five clear peaks along the average LOD profiles at the five predefined QTL positions. LOD scores around the predefined QTL positions increased by the increasing PVE. For example, PVE of Q1 and Q3 were 12.5 and 6.25%, and LOD scores at the two predefined positions were 15.86 and 8.15, respectively. Q1, Q2 and Q5, having the same *PVE* = 12.5%, had almost the same LOD score. Likewise, LOD_A_ and LOD_AE_ increased as the increase in *PVE*_*A*_ and *PVE*_*AE*_.

Detection powers of the predefined QTL were higher than 80%, and FDR was 14.11% (Table 6). The larger PVE was, the higher detection power would be. Q1, Q2 and Q5 had the same *PVE* = 12.5%, and their detection powers were 91.2, 98.3 and 89.5% respectively; the detection powers were 85.4 and 80.7% for Q3 and Q4, respectively, having the same PVE of 6.25%.

**Table 6:** Estimates of QTL positions and effects, and power analysis in the unlinked model based on 1000 simulation runs.

QTL	Pos. (cM) (mean±SE)	A (mean±SE)	AE_1_ (mean±SE)	AE_2_ (mean±SE)	Power (%)
Q1	16.21±2.33	0.47±0.07	0.00±0.07	0.00±0.07	91.2
Q2	2.83±2.01	0.00±0.06	0.47±0.06	-0.47±0.06	98.3
Q3	32.59±2.58	0.24±0.05	-0.24±0.05	0.24±0.05	85.4
Q4	26.16±2.62	0.24±0.06	0.24±0.05	-0.24±0.05	80.7
Q5	34.92±2.26	0.46±0.07	0.00±0.08	0.00±0.08	89.5
FDR = 14.11%					

A, AE_1_ and AE_2_ represent average additive effect, additive QEI effect in E_1_ and additive QEI effect in E_2_, respectively. Estimated positions and effects were calculated as the average of significant QTL in the support interval. Power of each QTL was calculated as the proportion of runs where the QTL was detected in the 10 cM support interval. FDR, false positive rate, was calculated as the proportion of false QTL to the total number of QTL detected. SE: standard error.

**Fig 3:**
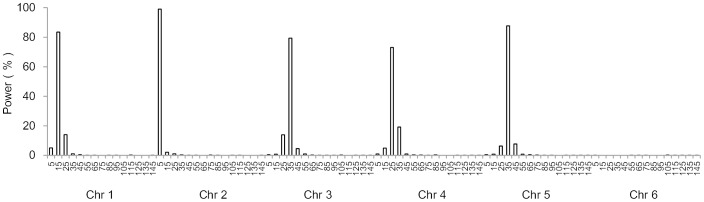
Power analysis by marker interval for the unlinked QTL model. Power was calculated as the proportion of runs where QTL on the interval was detected. There were 90 marker intervals defined by the 96 markers evenly distributed on six chromosomes.

When detection powers were calculated by marker intervals along the six chromosomes, it can be seen that most detected QTL were distributed around the marker intervals where the QTL were located (Fig 3). In other words, the ICIM QEI mapping was less likely to locate a QTL in chromosome regions far from the predefined QTL or in other chromosomes where no QTL were located. For example, Q1 was located at 16 cM on chromosome 1. Power at the marker interval where Q1 was located was 83.4%, and powers at its nearest left and right intervals were 5.1% and 14.0%, respectively. Powers at other intervals on chromosome 1 were close to 0.

### Estimation of QTL positions and effects for the unlinked model

Estimated QTL positions and effects from the 1000 simulated populations given in Table 6 showed their unbiasedness. Estimated positions of Q1 to Q5 were 16.21, 2.83, 32.59, 26.16 and 34.92 cM, corresponding to the true values 16, 3, 33, 26 and 35 cM on chromosomes 1 to 5. Estimated average additive effects of Q1 to Q5 were 0.47, 0.00, 0.24, 0.24 and 0.46, corresponding to the true values 0.5, 0, 0.25, 0.25 and 0.5 respectively. Estimated QEI effects in E_1_ were 0.00, 0.47, -0.24, 0.24 and 0.00, corresponding to the true values 0, 0.5, -0.25, 0.25 and 0 respectively. Estimated QEI effects in E_2_ were 0.00, -0.47, 0.24, -0.24 and 0.00, corresponding to the true effects 0, -0.5, 0.25, -0.25 and 0 respectively. The standard errors of the estimated positions and effects across 1000 simulations ranged from 2.01 to 2.62 and from 0.05 to 0.08, respectively.

### Power analysis for the linked QTL model

**Fig 4:**
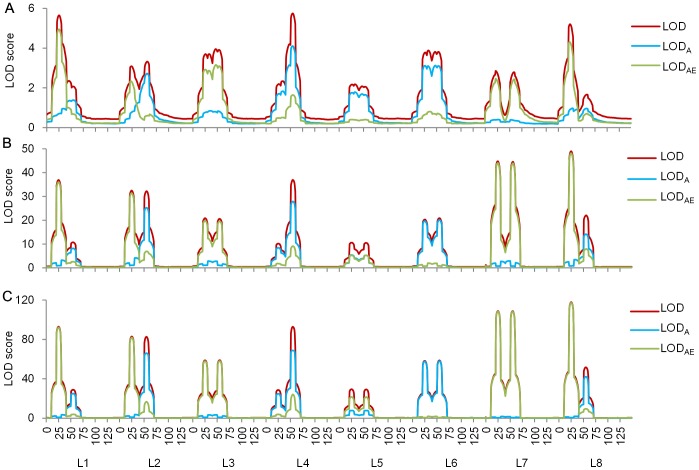
Average LOD profiles on chromosome 1 across 1000 simulation runs for the linked QTL model. (A) *H* ^2^ = 0.1. (B) *H* ^2^ = 0.5. (C) *H* ^2^ = 0.8. LOD scores on other chromosomes were not shown because no QTL was defined there. LOD score was close to zero on chromosomes 2 to 6.

Fig 4 showed average LOD score profiles of the eight effect models senarios under three heritability levels. Clear peaks were observed around the predefined positions of the two linked QTL, especially for higher heritabilities 0.5 and 0.8 (Fig 4B and 4C). The trend that higher PVE resulted in larger LOD score was also observed in linked QTL model. For example, PVE of Q1 and Q2 in models L3 and L6 were both 16.14% under *H* ^2^ = 0.5, and similar LOD scores at peaks around predefined positions were obtained, i.e., 20.84 and 20.60 in model L3, and 20.32 and 20.85 in model L6, respectively. PVE of Q1 and Q2 in model L2 were both 25% under *H* ^2^ = 0.5, and the LOD scores at peaks around QTL were 32.46 and 32.22, respectively, which were much higher than those in models L3 and L6 (Fig 4B).

For the same QTL effect model, higher heritability results in larger PVE and consequently increases the LOD score. Compared with *H* ^2^ = 0.1, LOD scores at QTL peaks of *H* ^2^ = 0.5 were larger for all effect models. LOD scores at QTL peaks of *H* ^2^ = 0.8 were the largest for the three heritability levels (Fig 4). For instance, LOD scores at peaks around Q1 and Q2 in model L8 were 5.20 and 1.67 for *H* ^2^ = 0.1, 48.99 and 22.01 for *H* ^2^ = 0.5, and 117.96 and 51.75 for *H* ^2^ = 0.8. Linked QTL with larger PVE were also easier to be separated. For example for *H* ^2^ = 0.1, PVE of Q1 and Q2 in model L1 were 4.88 and 2.44%, and only one peak appeared in the average LOD profile (Fig 4A). PVE of Q1 and Q2 in model L7 were both 11.08% for *H* ^2^ = 0.1, and two clear peaks appeared around the two predefined QTL positions.

**Fig 5:**
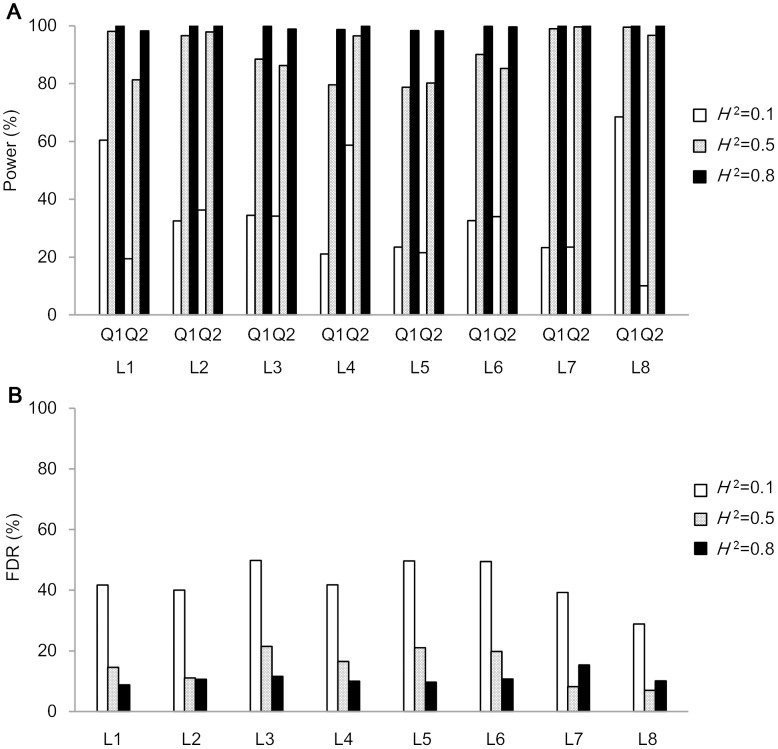
Power (A) and FDR (B) for linked QTL model. Power (A) of each QTL was calculated as the proportion of runs where the QTL was identified in a 10 cM support interval. FDR (B), false discovery rate, was calculated as the proportion of false positive QTL to total QTL detected for each model and each heritability level.

Fig 5 displayed the detection power and FDR for the two linked QTL in QEI mapping. Detection power increased with the increase of heritability and PVE of QTL. For example in model L1 under *H* ^2^ = 0.1, 0.5 and 0.8, PVE of Q1 were 4.88, 24.40 and 39.05%, and PVE of Q2 were 2.44, 12.20 and 19.52%, respectively. Their detection powers were 60.4 and 19.4% for *H* ^2^ = 0.1, 98.1 and 81.3% for *H* ^2^ = 0.5, and 100 and 98.2% for *H* ^2^ = 0.8. FDR decreased by the increase of heritability. Taking model L1 as an example, FDR were 41.7, 14.7 and 8.8% for *H* ^2^ = 0.1, 0.5 and 0.8, respectively. Powers of all QTL in all effect models increased to about 80% for *H* ^2^ = 0.5 and nearly 100% for *H* ^2^ = 0.8. Meanwhile, FDR of all effect models was less than 22% for *H* ^2^ = 0.5 and less than 16% for *H* ^2^ = 0.8.

**Fig 6:**
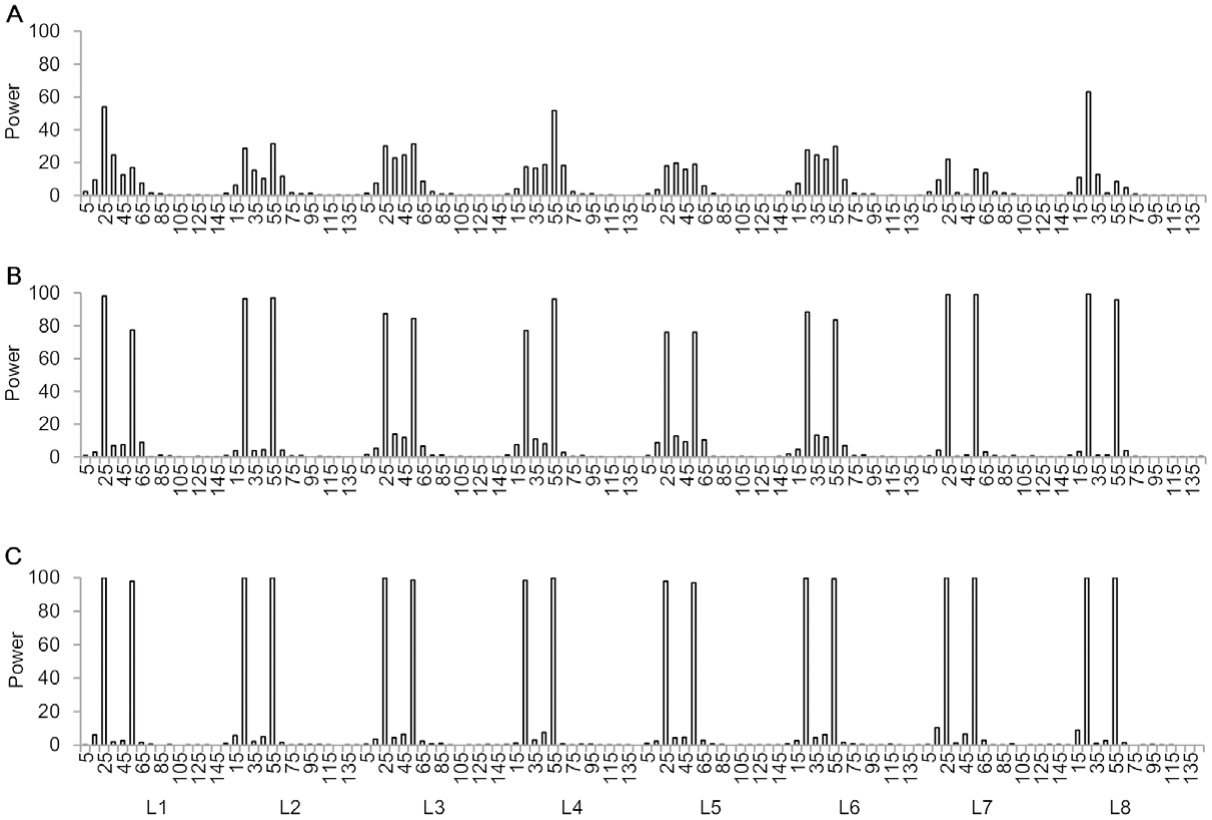
Power analysis by marker interval of chromosome 1 for the linked QTL model. (A) *H* ^2^ = 0.1. (B) *H* ^2^ = 0.5. (C) *H* ^2^ = 0.8. Power was calculated as the proportion of runs where QTL on the interval was detected. There were 15 marker intervals defined by the 16 markers evenly distributed on chromosome 1. Power on other chromosomes were close to zero on chromosomes 2 to 6 where no QTL was predefined.

When detection powers were calculated by marker intervals along chromosome 1, most detected QTL were distributed around the marker intervals where the two QTL were located especially for *H* ^2^ = 0.5 and *H* ^2^ = 0.8 (Fig 6). For example, Q1 and Q2 in model L3 were located at 25 and 55 cM on chromosome 1. Powers at their two marker intervals were 96.4 and 96.9%, respectively for *H* ^2^ = 0.5. Powers were 3.8 and 3.7% at the nearest left and right intervals of Q1, and 4.4 and 4.1% at the nearest left and right intervals of Q2. Powers were rather low at other intervals. It could be found that even for the linked QTL, ICIM QEI mapping was less likely to locate a QTL in chromosome regions far away from the predefined QTL or in other chromosomes where no QTL were located.

### QEI mapping in the maize RIL population

**Fig 7:**
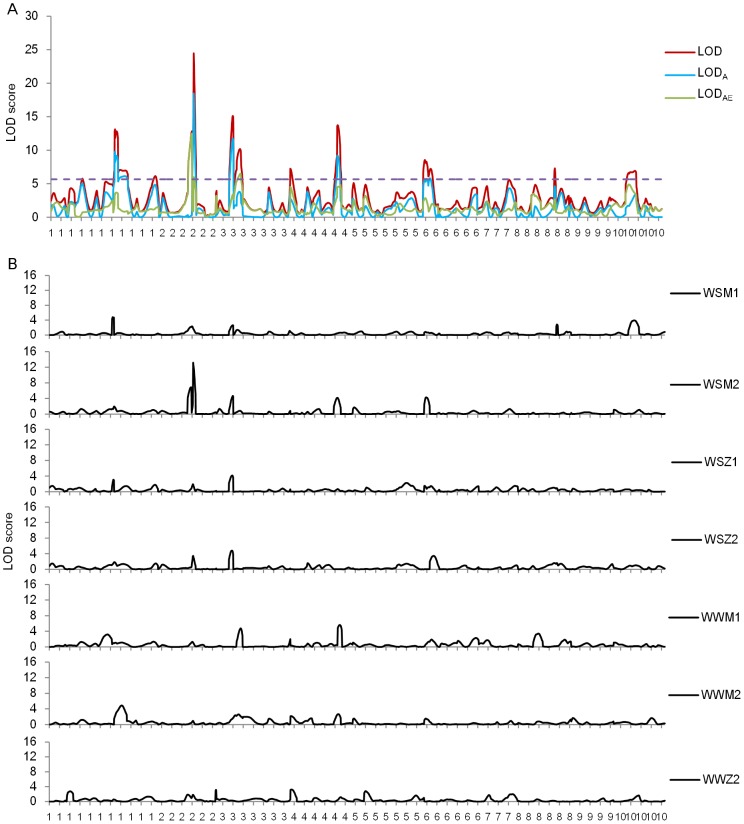
LOD profiles for MFLW in the maize population by QEI mapping (A) and single-environment analysis (B). The dash line denotes the LOD threshold of 5.67 in QEI mapping.

Through stepwise regression, 4, 5, 2, 3, 4, 2 and 4 markers were selected for environments WSM1, WSM2, WSZ1, WSZ2, WWM1, WWM2 and WWZ2 respectively. In QEI mapping, profiles of LOD, LOD_A_ and LOD_AE_ along the maize genome were shown in Fig 7A. Under the LOD threshold 5.67, a total of 13 QTL affecting MFLW were identified across the seven environments: one each on chromosomes 8 and 10, two each on chromosomes 2, 3, 4, and 6, and three on chromosome 1 (Table 7). Although QEI was observed in some chromosomal regions, e.g., chromosomes 2, 3 and 10 (Fig 7A), most identified QTL were relatively stable with large LOD_A_ and small LOD_AE_ (Table 7). Five QTL had positive average effects (Table 7). *qMFLW-2-2* had the highest LOD = 24.45, LOD_A_ = 18.45 and LOD_AE_ = 5.99, and had the largest average and QEI effects as well. Average additive effect was -0.43, indicating the allele from CML444 at this locus would reduce MFLW by 0.43 days on the basis of population mean. *qMFLW-1-1* was relatively stable, whose LOD = 5.76, LOD_A_ = 5.07 and LOD_AE_ = 0.70. The allele of CML444 at this locus would delay MFLW by 0.35 days. *qMFLW-2-1* had strong QEI, whose LOD = 12.82, LOD_A_ = 0.31 and LOD_AE_ = 12.50.

**Table 7:** Estimated effects and positions of QTL detected in the maize RIL population by QEI mapping.

QTL	Chr.	Pos.	Left Marker	Right Marker	LOD	LOD_A_	LOD_AE_	PVE (%)	PVE_A_ (%)	PVE_AE_ (%)	Additive effect in each environment							Average effect	Additive QEI effect						
											WSM1	WSM2	WSZ1	WSZ2	WWM1	WWM2	WWZ2		WSM1	WSM2	WSZ1	WSZ2	WWM1	WWM2	WWZ2
*qMFLW-1-1*	1	108	bnlg439(03)	bnlg2238(04)	5.76	5.07	0.70	1.30	1.11	0.19	-0.06	-0.26	-0.32	-0.23	-0.26	-0.31	-0.11	-0.22	0.16	-0.04	-0.10	0.00	-0.04	-0.09	0.11
*qMFLW-1-2*	1	221	umc128(08)	umc166b(08)	13.14	9.80	3.34	3.06	2.16	0.90	0.53	0.31	0.58	0.41	0.23	-0.01	0.11	0.31	0.22	0.00	0.27	0.10	-0.08	-0.31	-0.20
*qMFLW-1-3*	1	360	bnlg2331(11)	bnlg2123(11)	6.14	4.85	1.30	1.44	0.99	0.45	0.05	-0.22	-0.41	-0.34	-0.25	-0.21	-0.09	-0.21	0.26	-0.01	-0.20	-0.13	-0.04	0.00	0.12
*qMFLW-2-1*	2	114	umc135(04)	umc8g(05)	12.82	0.31	12.50	2.44	0.07	2.37	-0.34	0.62	-0.32	0.21	-0.24	-0.18	-0.15	-0.06	-0.28	0.68	-0.26	0.27	-0.19	-0.12	-0.09
*qMFLW-2-2*	2	120	csu54a(07)	umc55a(06)	24.45	18.45	5.99	5.39	4.18	1.21	-0.31	-0.90	-0.47	-0.60	-0.24	-0.27	-0.21	-0.43	0.12	-0.47	-0.04	-0.17	0.19	0.16	0.22
*qMFLW-3-1*	3	55	bnlg1447(03)	umc154(04)	15.11	11.73	3.39	4.39	2.64	1.74	0.05	0.51	0.72	0.72	0.02	0.35	0.08	0.35	-0.30	0.16	0.37	0.37	-0.33	0.00	-0.27
*qMFLW-3-2*	3	80	bnlg1019a(04)	phi053(05)	10.18	3.73	6.46	1.80	0.75	1.05	0.38	0.21	-0.07	-0.08	0.49	0.43	-0.01	0.19	0.19	0.02	-0.26	-0.27	0.29	0.24	-0.20
*qMFLW-4-1*	4	0	umc1017(01)	umc1294(02)	7.24	2.72	4.51	1.15	0.61	0.55	-0.27	-0.02	-0.06	0.05	-0.14	-0.42	-0.28	-0.16	-0.11	0.14	0.11	0.21	0.02	-0.26	-0.11
*qMFLW-4-2*	4	164	csu11b	npi593a(09)	13.73	9.17	4.57	2.70	2.00	0.69	0.21	0.45	0.27	0.04	0.54	0.46	0.11	0.30	-0.08	0.16	-0.03	-0.26	0.24	0.16	-0.19
*qMFLW-6-1*	6	6	umc85a(01)	bnlg426(01)	8.54	5.73	2.80	1.67	1.15	0.52	-0.23	-0.47	-0.33	-0.03	-0.19	-0.33	-0.01	-0.23	-0.01	-0.24	-0.10	0.20	0.04	-0.11	0.22
*qMFLW-6-2*	6	26	umc36c(01)	bnlg2151(02)	7.23	5.64	1.59	1.77	1.14	0.63	-0.22	-0.05	-0.34	-0.54	-0.29	-0.12	-0.02	-0.23	0.01	0.18	-0.12	-0.31	-0.06	0.10	0.20
*qMFLW-8*	8	133	umc48a(06)	asg52a(06)	7.29	4.63	2.66	1.72	1.03	0.69	0.49	0.08	0.34	0.41	0.11	0.06	0.02	0.21	0.27	-0.14	0.12	0.19	-0.11	-0.15	-0.19
*qMFLW-10*	10	82	bnlg1079(03)	umc1115(04)	6.91	3.35	3.56	1.09	0.66	0.42	-0.38	-0.20	-0.20	-0.28	0.08	-0.06	-0.18	-0.17	-0.21	-0.03	-0.02	-0.11	0.26	0.11	0.00

Values underlined indicate the common QTL detected by both QEI mapping and single-environment analysis. Positive effect indicates the allele increasing trait value is from parental line CML444. Negative effect indicates the allele increasing trait value is from parental line SC-Malawi. PVE, PVE_A_ and PVE_AE_ are the percentages of variance explained by all effects, major effect and QEI effect of the detected QTL respectively.

LOD profiles along the maize genome by single-environment analysis were shown in Fig 7B. Under the LOD threshold 3.0, a total of 19 QTL were identified, four of which were coincident in more than two environments. Respectively, 2, 5, 2, 3, 4, 1 and 2 QTL were identified in environments WSM1, WSM2, WSZ1, WSZ2, WWM1, WWM2 and WWZ2 (Table 8). Ten QTL detected by QEI mapping, i.e., *qMFLW-1-2*, *qMFLW-2-1*, *qMFLW-2-2*, *qMFLW-3-1*, *qMFLW-3-2*, *qMFLW-4-1*, *qMFLW-4-2*, *qMFLW-6-1*, *qMFLW-6-2* and *qMFLW-10*, were also detected by single-environment analysis, while other QTL were detected only by single-environment analysis (Tables 7 and 8). Take a common QTL around 220 cM on chromosome 1 as an example, the estimated positions were 217 and 220 cM by single-environment analysis in WSM1 and WSZ1 respectively, which was close to *qMFLW-1-2* detected by QEI mapping. Compared with single-environment analysis, QEI mapping has the following properties.

**Table 8:** Estimated effects and positions of QTL detected in the maize RIL population by single-environment analysis.

Environment	Chr.	Pos.	Left Marker	Right Marker	LOD	PVE	Add	QTL identified by QEI mapping
WSM1	1	217	umc1128(07)	umc128(08)	4.8	9.71	0.66	*qMFLW-1-2*
WSM1	10	70	bnlg1079(03)	umc1115(04)	3.94	11.07	-0.7	*qMFLW-10*
WSM2	2	113	umc135(04)	umc8g(05)	6.85	11.52	0.67	*qMFLW-2-1*
WSM2	2	120	csu54a(07)	umc55a(06)	13.17	21.93	-0.92	*qMFLW-2-2*
WSM2	3	56	umc154(04)	umc92a(04)	4.66	7.29	0.55	*qMFLW-3-1*
WSM2	4	161	umc15a(08)	csu11b	4.16	6.59	0.5	*qMFLW-4-2*
WSM2	6	6	umc85a(01)	bnlg426(01)	4.3	7.36	-0.54	*qMFLW-6-1*
WSZ1	1	220	umc128(08)	umc166b(08)	3.05	5.78	0.64	*qMFLW-1-2*
WSZ1	3	54	bnlg1447(03)	umc154(04)	4.14	8.24	0.79	*qMFLW-3-1*
WSZ2	2	120	csu54a(07)	umc55a(06)	3.46	6.04	-0.63	*qMFLW-2-2*
WSZ2	3	53	bnlg1447(03)	umc154(04)	4.79	9.78	0.81	*qMFLW-3-1*
WSZ2	6	32	bnlg2151(02)	umc1887(03)	3.42	7.14	-0.69	*qMFLW-6-2*
WWM1	1	199	umc1122(06)	umc1128(07)	3.18	9.1	0.59	*—*
WWM1	3	83	bnlg1019a(04)	phi053(05)	4.75	7.49	0.57	*qMFLW-3-2*
WWM1	4	169	csu11b	npi593a(09)	5.62	9.88	0.61	*qMFLW-4-2*
WWM1	8	69	bnlg669(03)	umc1858(04)	3.41	7.62	-0.54	*—*
WWM2	1	248	dupssr12(08)	phi011(09)	4.87	20.87	1.01	*—*
WWZ2	2	198	csu109a(09)	umc36a(09)	3.14	5.49	-0.29	*—*
WWZ2	4	4	umc1017(01)	umc1294(02)	3.23	7.21	-0.33	*qMFLW-4-1*

Positive effect indicates the allele increasing trait value is from parental line CML444. Negative effect indicates the allele increasing trait value is from parental line SC-Malawi. PVE is the percentage of variance explained by individual QTL.

1. Both QTL stability and QEI effect can be analyzed. For example, major effect of *qMFLW-1-1* was -0.22 and additive by environment effects in the seven environments were 0.16, -0.04, -0.10, 0.00, -0.04, -0.09, and 0.11 respectively. It could be considered to be stable, as the absolute value of major effect was much larger than the interactions. In contrary, major effect of *qMFLW-2-1* was -0.06, but additive by environment effects in the seven environments were -0.28, 0.68, -0.26, 0.27, -0.19, -0.12 and -0.09 respectively. The much larger interactions showed the higher level of QEI, and the less stability.
2. Most QTL identified by single-environment analysis can also be detected by QEI mapping, especially for QTL detected in more than one environment. QEI mapping can detect some QTL which were not identified by single-environment analysis. In this study, *qMFLW-1-1*, *qMFLW-1-3* and *qMFLW-8* were detected only by QEI mapping (Tables 7 and 8). Small peaks with LOD scores lower than threshold 3.0 were observed around these QTL positions on LOD profiles in several environments. Therefore, it is not strange that they were detected in QEI mapping (Fig 7). For example, *qMFLW-8* identified by QEI mapping located at 133 cM on chromosome 8. Peaks of LOD scores were also observed around this position in environments WSM1 and WSZ2 by single-environment analysis.
3. For some common QTL, positions estimated by single-environment analysis fluctuated around the positions estimated by QEI mapping. For example, *qMFLW-3-1* was located at 55 cM on chromosome 3 in QEI mapping, but was located at 56, 54 and 53 cM on chromosome 3 in WSM2, WSZ1 and WSZ2, respectively, by single-environment analysis (Tables 7 and 8). QEI mapping used multi-environment phenotypic data simultaneously and therefore may result in more precise and reliable estimation of QTL position.
4. The estimated effects by QEI mapping and single-environment analysis were similar, although minor differences were observed. Taking *qMFLW-4-2* as an example, the estimated effects were 0.50 and 0.61 in WSM2 and WWM1 by single-environment analysis, corresponding to 0.45 and 0.54 estimated by QEI mapping (Tables 7 and 8).

Compared with the mapping results in Messmer et al. [39], the ICIM QEI mapping detect more QTL. Four QTL reported by the joint analysis for seven environments in Messmer et al. [39] were all identified in this study, i.e., *qMFLW-1-2*, *qMFLW-3-1*, *qMFLW-4-2* and *qMFLW-6-1*. However, nine more QTL were detected by QEI mapping in this study, and many of which can also be detected in single-environment analysis. For example, LOD score of *qMFLW-2-2* was 24.45 by QEI mapping. It was identified in environments WSM2 and WSZ2 with LOD scores 13.17 and 3.46 by single-environment analysis. In addition, there were peaks at LOD profiles in environments WSZ1, WWM2 and WWZ2 lower than LOD threshold in single-environment analysis. Similar were *qMFLW-2-1*, *qMFLW-4-1* and *qMFLW-10*. The truth of these QTL was validated by the single-environment analysis.

## Discussion

QEI can be investigated when genetic populations are planted in multiple locations and/or years. QEI information thus obtained is of great value for breeders and genetic researchers. According to the QTL mapping results, breeders can design ideal genotype of favorable alleles and more efficiently perform marker-assisted selection. Stable QTL of agronomic traits is useful to a wide range of environments, while environment-favorable QTL can be used within specific target environments. However, the detection of QEI is not easy.

“Results of separate analysis by environment is hard to interpret, and cannot take advantage of built-in replication provided by multiple environments”, pointed by Tinker and Mather [8]. Single-environment analysis is subject to the errors from different environments probably resulting in different positions and effects of the same QTL. It is not inconvenient to evaluate QTL stability and QEI effect by directly comparing the effects estimated by single-environment analysis. Some studies conduct QTL mapping using the mean phenotypic value across multi-environments or the genotypic value predicted by best linear unbiased prediction [41,42]. But this approach can only detect QTL with significant major effects. When dealing with multi-environment phenotypic data, the estimated positions and effects by QEI mapping are more reliable than single-environment analysis as the data across all environments are used simultaneously.

The two-step strategy used in ICIM simplifies the mapping procedure by separating the cofactor selection from interval mapping [7]. This study demonstrated that the superiority of ICIM has been maintained when extended to QEI mapping. Stepwise regression was conducted only once, based on which the phenotype was adjusted during the interval mapping. This strategy avoids the repeated interval mapping in Boer et al. [1], and requires much less computing time. Another feature distinguishing ICIM from other methods is that major effect and QEI effect of QTL are estimated based on genotypic value of two QTL genotypes, *QQ* and *qq*, across multi-environments through the orthogonal decomposition. QTL stability and QEI level can be directly evaluated from the mapping results, including three LODs, three PVEs, major effects and QEI effects.

Using similar algorithms described in this study, ICIM has been extended to dominant QEI mapping, and epistatic QEI mapping as well. In the case of epistatic QEI mapping, the first step was to use stepwise regression to select significant markers and significant marker pairs in each environment. The second step was to apply the two-dimensional interval mapping on the adjusted phenotypic values. Both major epistasis effect and epistasis by environment interaction effect can be estimated. QEI mapping of additive, additive and dominant, and epistatic effects in most biparental populations have been well implemented in the QTL IciMapping software [37].

LOD threshold is used to control false positive in QTL mapping. Use of a suitable threshold is an important issue, as it determines the number of identified QTL and control the genome-wide error rate. For convenience, we used an empirical formula to select the LOD threshold in this study. The LOD threshold to define a stable QTL can also be obtained from the empirical formula. For LOD_A_, *df* is equal to 1 for DH, BC_1_ and RIL populations, and 2 for F_2_ population when one-dimension scanning is conducted. Similarly for LOD_AE_, *df* is equal to *e*-1 for DH, BC_1_ and RIL populations, and 2(*e*-1) for F_2_ populations to obtain LOD threshold for significant QEI.

LOD threshold in QEI mapping is much higher than that in single-environment analysis, due to the increased degree of freedom. Some QTL detected in single environment may result in peaks lower than the threshold, which will not be reported in QEI mapping. But, most QTL detected in more than one environment and QTL with higher LOD score in single-environment analysis are more likely to be detected in QEI mapping. In the maize RIL population, most QTL detected in WS condition had higher LOD than that in WW condition. For example, 12 QTL were detected in WS, 8 of which had LOD scores over 4. In WW condition, 7 QTL were detected, 3 of which had LOD scores over 4. In addition, more QTL in WS were detected in more than one environment. For example, QTL around 217 cM on chromosome 1 was detected in WSM1 and WSZ1; QTL around 120 cM on chromosome 2 was detected in WSM2 and WSZ2. In comparison, more QTL in WW were detected in only one environment, especially those not identified by QEI mapping.

Genetic architecture of quantitative traits could be more complicated than additive, dominance and digenic epsitatsis discussed in this study. The environments where the traits are phenotyped can be equally complicated. Though we see benefits to apply QEI analysis for multi-environmental trials, we cannot exclude the use of QTL mapping by each environment, and then summarize the mapping results across the environments. Neither can we exclude the use of the estimated breeding values in QTL mapping where the target is to locate the highly-adapted and stable genes.

## Supporting Information

S1 File Input files for simulating BC_1_ populations under null genetic model and the 1000 simulated BC_1_ populations to validate empirical LOD threshold formula under 1, 2, 3, 4, 5, 6, 8 and 10 environments, respectively. (ZIP)

S2 File Input files for simulating F_2_ populations under null genetic model and the 1000 simulated F_2_ populations to validate empirical LOD threshold formula under 1, 2, 3, 4, 5, 6, 8 and 10 environments, respectively. (ZIP)

S3 File Input files for simulatiing DH populations under unlinked genetic model and the 1000 simulated DH populations. (ZIP)

S4 File Input files for simulating DH populations under linked genetic models (L1-L8) with *H* ^2^ = 0.1 and the 1000 simulated DH populations. (ZIP)

S5 File Input files for simulating DH populations under linked genetic models (L1-L8) with *H* ^2^ = 0.5 and the 1000 simulated DH populations. (ZIP)

S6 File Input files for simulating DH populations under linked genetic models (L1-L8) with *H* ^2^ = 0.8 and the 1000 simulated DH populations. (ZIP)

S7 File Input files for maize RIL population used in single-environment analysis and QEI mapping. (ZIP)

S8 File QTL identified in the 1000 simulated DH populations under unlinked genetic model. (ZIP)

S9 File Values to build all figures and tables in the paper. (XLSX)

## References

[1] Boer MP, Wright D, Feng L, Podlich DW, Luo L, Cooper M, et al A mixed-model quantitative trait loci (QTL) analysis for multiple-environment trial data using environmental covariables for QTL-by-environment interactions, with an example in maize. Genetics. 2007; 177: 1801–1813. 17947443 10.1534/genetics.107.071068 PMC2147942

[2] Collard BCY, Mackill DJ. Marker-assisted selection: an approach for precision plant breeding in the twenty-first century. Phil Trans R Soc B. 2008; 363: 557–572. 17715053 10.1098/rstb.2007.2170 PMC2610170

[3] EI-Soda M, Malosetti M, Zwaan BJ, Koornneef M, Aarts MGM. Genotype× environment interaction QTL mapping in plants: lessons from Arabidopsis. Trends Plant Sci. 2014; 19: 390–398. 10.1016/j.tplants.2014.01.001 24491827

[4] Pillen K, Zacharias A, Léon J. Advanced backcross QTL analysis in barley (*Hordeum vulgare* L). Theor Appl Genet. 2003; 107: 340–352. 12677407 10.1007/s00122-003-1253-9

[5] Jiang C, Zeng Z. Multiple trait analysis of genetic mapping for quantitative trait loci. Genetics. 1995; 140: 1111–1127. 7672582 10.1093/genetics/140.3.1111 PMC1206666

[6] Jansen RC, Van Ooijen JW, Stam P, Lister C, Dean C. Genotype-by-environment interaction in genetic mapping of multiple quantitative trait loci. Theor Appl Genet. 1995; 91: 33–37. 10.1007/BF00220855 24169664

[7] Li H, Ye G, Wang J. A modified algorithm for the improvement of composite interval mapping. Genetics. 2007; 175: 361–374. 17110476 10.1534/genetics.106.066811 PMC1775001

[8] Tinker NA, Mather DE. Methods for QTL analysis with progeny replicated in multiple environments. J Quantitative Trait Loci. 1995; Avaliable: http://wheat.pw.usda.gov/jag/papers95/paper195/jqtl15.html.

[9] Korol AB, Ronin YI, Nevo E. Approximate analysis of QTL-environment interaction with no limits on the number of environments. Genetics. 1998; 148: 2015–2028. 9560414 10.1093/genetics/148.4.2015 PMC1460115

[10] Hackett CA, Meyer RC, Thomas WTB. Multi-trait QTL mapping in barley using multivariate regression. Genet Res (Camb). 2001; 77: 95–106. 10.1017/s0016672300004869 11279835

[11] Haley CS, Knott SA. A simple regression method for mapping quantitative trait loci in line crosses using flanking markers. Heredity. 1992; 69: 315–324. 16718932 10.1038/hdy.1992.131

[12] Wang DL, Zhu J, Li ZK, Paterson AH. Mapping QTLs with epistatic effects and QTL×environment interactions by mixed linear model approaches. Theor Appl Genet. 1999; 99: 1255–1264.

[13] Piepho H. A mixed-model approach to mapping quantitative trait loci in barley on the basis of multiple environment data. Genetics. 2000; 156: 2043–2050. 11102394 10.1093/genetics/156.4.2043 PMC1461386

[14] Malosetti M, Voltas J, Romagosa I, Ullrich SE, van Eeuwijk FA. Mixed models including environmental covariables for studying QTL by environment interaction. Euphytica. 2004; 137: 139–145.

[15] Vargas M, van Eeuwijk FA, Crossa J, Ribaut J. Mapping QTLs and QTL×environment interaction for CIMMYT maize drought stress program using factorial regression and partial least squares methods. Theor Appl Genet. 2006; 112: 1009–1023. 16538513 10.1007/s00122-005-0204-z

[16] Crossa J, Vargas M, van Eeuwijk FA, Jiang C, Edmeades GO, Hoisington D. Interpreting genotype×environment interaction in tropical maize using linked molecular markers and environmental covariables. Theor Appl Genet. 1999; 99: 611–625. 10.1007/s001220051276 22665197

[17] van Eeuwijk FA, Crossa J, Vargas M, Ribaut J. Analysing QTL-environment interaction by factorial regression, with an application to the CIMMYT drought and low-nitrogen stress programme in maize In: Kang MS (ed) Quantitative genetics, genomics and plant breeding. Wallingford: CABI; 2002 pp. 245–256.

[18] Chen X, Zhao F, Xu S. Mapping environment-specific quantitative trait loci. Genetics. 2010; 186: 1053–1066. 10.1534/genetics.110.120311 20805558 PMC2975292

[19] Zhao F, Xu S. Genotype by environment interaction of quantitative traits: a case study in barley. G3. 2012; 2: 779–788. 10.1534/g3.112.002980 22870401 PMC3385984

[20] Gauch HG Jr, Rodrigues PC, Munkvold JD, Heffner EL, Sorrell M. Two new strategies for detecting and understanding QTL×environment interactions. Crop Sci. 2011; 51: 96–113.

[21] Wang J, Li H, Zhang L. Genetic Mapping and Breeding Design. 1st ed Beijing: Science Press; 2014.

[22] Li H, Ribaut J, Li Z, Wang J. Inclusive composite interval mapping (ICIM) for digenic epistasis of quantitative traits in populations. Theor Appl Genet. 2008; 116: 243–260. 17985112 10.1007/s00122-007-0663-5

[23] Zhang L, Li H, Li Z, Wang J. Interactions between markers can be caused by the dominance effect of quantitative trait loci. Genetics. 2008; 180: 1177–1190. 10.1534/genetics.108.092122 18780741 PMC2567366

[24] Wang J. Inclusive composite interval mapping of quantitative trait genes. Acta Agron Sin. 2009; 35: 239–245 (in Chinese with English abstract).

[25] Wu W, Li W, Lu H. A general approach for filtrating genetic background noise in QTL mapping. Journal of Biomathematics. 1998; 13: 592–595.

[26] Li H, Zhang L, Wang J. Estimation of statistical power and false discovery rate of QTL mapping methods through computer simulation. Chin Sci Bull. 2012; 57: 2701–2710.

[27] Zhang L, Li H, Wang J. The statistical power of inclusive composite interval mapping in detecting digenic epistasis showing common F_2_ segregation ratios. J Integr Plant Biol. 2012; 54: 270–279. 10.1111/j.1744-7909.2012.01110.x 22348947

[28] Alves AA, Rosado CCG, Faria DA, Guimarães LMS, Lau D, Brommonschenkel SH, et al Genetic mapping provides evidence for the role of additive and non-additive QTLs in the response of inter-specific hybrids of *Eucalyptus* to *Puccinia psidii* rust infection. Euphytica. 2012; 183: 27–38.

[29] Li X, Chen X, Xiao Y, Xia X, Wang D, He Z, et al Identification of QTLs for seedling vigor in winter wheat. Euphytica. 2014; 198: 199–209.

[30] Njau PN, Bhavani S, Huerta-Espino J, Keller B, Singh RP. Identification of QTL associated with durable adult plant resistance to stem rust race Ug99 in wheat cultivar ‘Pavon 76’. Euphytica. 2013; 190: 33–44.

[31] Yuste-Lisbona FJ, Capel C, Sarria E, Torreblanca R, Gόmez-Guillamόn ML, Capel J, et al Genetic linkage map of melon (*Cucumis melo* L) and localization of a major QTL for powdery mildew resistance. Mol Breed. 2011; 27: 181–192.

[32] Zeng Z. Precision mapping of quantitative trait loci. Genetics. 1994; 136: 1457–1468. 8013918 10.1093/genetics/136.4.1457 PMC1205924

[33] Whittaker JC, Thompson R, Visscher PM. On the mapping of QTL by regression of phenotype on marker-type. Heredity. 1996; 77: 23–32.

[34] Meng X, Rubin DB. Maximum likelihood estimation via the ECM algorithm: a general framework. Biometrika. 1993; 80: 67–278.

[35] Piepho HP. A quick method for computing approximate thresholds for quantitative trait loci detection. Genetics. 2001; 157: 425–432. 11139522 10.1093/genetics/157.1.425 PMC1461497

[36] Sun Z, Li H, Zhang L, Wang J. Properties of the test statistic under null hypothesis and the calculation of LOD threshold in quantitative trait loci (QTL) mapping. Acta Agron Sin. 2013; 39: 1–11 (in Chinese with English abstract).

[37] Meng L, Li H, Zhang L, Wang J. QTL IciMapping: Integrated software for genetic linkage map construction and quantitative trait locus mapping in biparental populations. The Crop Journal 2015; in press.

[38] Li H, Hearne S, Bänziger M, Li Z, Wang J. Statistical properties of QTL linkage mapping in biparental genetic populations. Heredity. 2010; 105: 257–267. 10.1038/hdy.2010.56 20461101

[39] Messmer R, Fracheboud Y, Bänziger M, Vargas M, Stamp P, Ribaut J. Drought stress and tropical maize: QTL-by-environment interactions and stability of QTLs across environments for yield components and secondary traits. Theor Appl Genet. 2009; 119: 913–930. 10.1007/s00122-009-1099-x 19597726

[40] Churchill GA, Doerge RW. Empirical threshold values for quantitative trait mapping. Genetics. 1994; 138: 963–971. 7851788 10.1093/genetics/138.3.963 PMC1206241

[41] Qin Y, Liu R, Mei H, Zhang T, Guo W. QTL Mapping for Yield Traits in Upland Cotton (*Gossypium hirsutum* L). Acta Agron Sin. 2009; 35: 1812–1821 (in Chinese with English abstract).

[42] Li Q, Yang X, Xu S, Cai Y, Zhang D, Han Y, et al Genome-wide association studies identified three independent polymorphisms associated with α-tocopherol content in maize kernels. PLoS One 2012 5 15; 7(5). 10.1371/journal.pone.0036807 PMC3352922 22615816

